# Characterization of the Flavors and Organoleptic Attributes of Petit Manseng Noble Rot Wines from the Eastern Foothills of Helan Mountain in Ningxia, China

**DOI:** 10.3390/foods14152723

**Published:** 2025-08-04

**Authors:** Fuqi Li, Fan Yang, Quan Ji, Longxuan Huo, Chen Qiao, Lin Pan

**Affiliations:** Ningxia Key Laboratory of Characteristic Resources Food and Biological Manufacturing, College of Food Science and Engineering, Ningxia University, Yinchuan 750021, China; 15595421925@163.com (F.L.); 17711866516@163.com (F.Y.); jq18295154870@163.com (Q.J.); 18966983021@163.com (L.H.); qiaochen322@163.com (C.Q.)

**Keywords:** gray mold, noble rot character, Petit Manseng, sweet white wines

## Abstract

To investigate the effect of *Botrytis cinerea* infection severity on the flavor characteristics of Petit Manseng noble rot wine, this study analyzed wines produced from Petit Manseng grapes grown in the eastern foothills of Helan Mountain, Ningxia, China. The grapes were categorized into three groups based on infection status: uninfected, mildly infected, and severely infected with *Botrytis cinerea*. Headspace solid-phase microextraction coupled with gas chromatography-mass spectrometry (HS-SPME-GC-MS) and an electronic nose were employed to detect and analyze the aroma components of wines under the three infection conditions. Additionally, trained sensory panelists conducted sensory evaluations of the wine aromas. The results revealed that wines made from severely infected grapes exhibited the richest and most complex aroma profiles. A total of 70 volatile compounds were identified, comprising 32 esters, 17 alcohols, 5 acids, 8 aldehydes and ketones, 4 terpenes, and 4 other compounds. Among these, esters and alcohols accounted for the highest contents. Key aroma-active compounds included isoamyl acetate, ethyl decanoate, phenethyl acetate, ethyl laurate, hexanoic acid, linalool, decanoic acid, citronellol, ethyl hexanoate, and methyl octanoate. Sensory evaluation indicated that the “floral aroma”, “pineapple/banana aroma”, “honey aroma”, and “overall aroma intensity” were most pronounced in the severely infected group. These findings provide theoretical support for the harvesting of severely *Botrytis cinerea*-infected Petit Manseng grapes and the production of high-quality noble rot wine in this region.

## 1. Introduction

Noble rot wine is a sweet wine made from grapes that have been botrytized with the beneficial fungus *B. cinerea*, also called noble rot. The process of botrytization is highly complex, involving both enzymatic transformations and the dehydration and concentration of the grape berries [1]. This transformation significantly elevates the concentrations of sugar, acidity, glycerol, minerals, and specific aroma compounds within the grapes.

During botrytization, the grapes’ inherent aromatic compounds are not only concentrated but also rendered more readily extractable from the skins during vinification. Consequently, botrytized wines exhibit a greater abundance of volatile aroma compounds compared to non-botrytized dry white wines [2]. Key compounds include furan derivatives (such as 5-hydroxymethylfurfural and sotolon), phenylacetaldehyde, and acetaldehyde. Furthermore, mercaptans [3] and ethyl esters of 2-, 3-, and 4-methylpentanoic acid are characteristic constituents [4]. These distinctive aromatic components contribute to the complex and captivating sensory profile of noble rot wine. The eastern foothills of Helan Mountain (Ningxia, China) are located in a “golden” wine grape growing belt at 38° N latitude. Due to the mountain barrier and the Yellow River irrigation system, this area has become important for producing high-quality wine in China [5]. Key features include more than 3000 h of sunshine per year and a significant difference in temperature between day and night (10–15 °C). In addition to cultivating international varieties of grapes, the region has explored the brewing potential of special varieties, such as Petit Manseng. This variety can be used to produce high-quality sweet wines because it ripens late (harvesting can be delayed until the end of November), maintains high sugar and acidity levels, and exhibits strong resistance to disease. However, gray mold infestation has been observed as spots on the surface of fruits during the ripening period. As pathogenic bacteria continue to proliferate during ripening, infested parts may crumple, and the skin of diseased fruits can become shriveled. However, the flesh tissue is usually free from lesions and retains its high concentration of sugars. These characteristics are typical of noble rot grapes used for brewing, and Ningxia has the conditions necessary for brewing noble rot wines [6]. However, a better understanding of how *Botrytis cinerea* infestation affects the flavor of wines produced from Petit Manseng grapes grown in Ningxia is needed. The aim of this study is to systematically investigate the effects of different degrees of noble rot (Botrytis cinerea) infestation (no infestation, mild infestation, severe infestation) on the flavor of Petit Manseng wines from the Helan Mountain East Foothill region, through identifying and quantifying volatile aroma compounds in wines produced from grapes with varying infestation degrees using HS-SPME-GC-MS and electronic nose, evaluating sensory characteristics (e.g., floral, fruity, honey notes) via trained panelists, determining the key aroma compounds associated with superior flavor in these wines, and providing a scientific basis for optimizing harvest and winemaking strategies for high-quality Petit Manseng noble rot wines in this region.

## 2. Materials and Methods

### 2.1. Grape Material

Petit Manseng grapes were sourced from the Helan Mountain Eastern Foothills Winery located in Yinchuan, Ningxia, China (approximately 3828′ N, 1024′ E). The vines were 8 years old, with single diagonal fencing and 0.3 m spacing. They yielded approximately 600 kg/mu (1500 kg/ha) of grapes. Uninfected healthy grapes, serving as the control group, were harvested on October 25; grapes with mild infection, characterized by approximately 11–50% of the berry surface presenting noble rot features, were collected on October 30; and grapes with severe infection, exhibiting over 51% surface coverage by *Botrytis cinerea* along with typical dehydration and shriveling, were harvested on November 5. These dates were chosen to ensure that the grape juice had the required sugar content and the weather was suitable for harvesting.

### 2.2. Chemicals and Reagents

Sodium chloride was obtained from Xuzhou Tianhong Chemical Company (Jiangsu Sheng, China). The glycerol kit used was from BioSystems (Barcelona, Spain). 4-methyl-2-pentanol (≥98.0% GC) was obtained from CI Division (Tokyo, Japan). Phenolphthalein and sulfuric acid were obtained from Tianjin Zhiyuan Chemical Reagent Company (Tianjin, China). Glucose was obtained from Tianjin Daimao Chemical Reagent Factory (Tianjin, China). Food-grade citric acid and sugar were from Rizhao Jinhe Boyuan Biochemical Company (Rizhao City, China). Analytical grade folinol was obtained from Beijing Solebao Technology Company (Beijing, China). Chromatography-grade p-dimethylcinnamic acid, catechin, and gallic acid were obtained from Shanghai Yuanye Biotechnology Company (Shanghai, China). C8–C20 n-alkanes (≥99.70% GC) were from Sigma-Aldrich (St. Louis, MO, USA). The active dry yeast Excellence XR was supplied by Lamothe-Abiet (Bordeaux, France; code LSA7). Vinozym vintage free-of-cinnamyl-esterase-activity pectinase and potassium metabisulfite were obtained from Lamothe-Abiet (Canéjan, France).

### 2.3. Instruments

The gas chromatograph (model 7890B), triple quadrupole mass spectrometer (model 7000D), chromatography column (30 m × 250 µm, 0.25 µm), DB-WAX, extraction head (50/30 µm, 1 cm), and divinylbenzene/carboxen/polydimethylsiloxane (DVB/CAR/PDMS) used in this study were all from Agilent Technologies (Santa Clara, CA, USA). The automated headspace injection GTC-PAL unit was from CTC Analytics (Zwingen, Switzerland). The spectrophotometer (model WSF) and precision pH meter (model PHS-3C) were from Yidian Instrument Company (Shanghai, China). The electronic nose (model PEN3.0) was from Airsense Analytics (Schwerin, Germany). The automatic wine analyzer (model Y15) was from BioSystems. The ultraviolet spectrophotometer (model UV-2700) was from Mettler Toledo (Greifensee, Switzerland).

### 2.4. Test Methods

#### 2.4.1. Petit Manseng Sweet Wines

**Figure 1:**
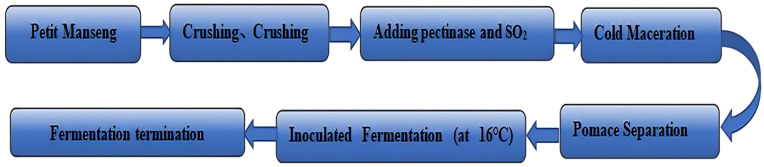
The flow chart of wine fermentation.

Harvested Petit Manseng grapes were separated into the following three groups: control group A grapes, with no infested grapes; lightly infested group B grapes, with black spots on approximately 11–50% of grapes; and severely infested group C grapes, with black spots on approximately 51–100% of grapes. The grapes from all three groups were from the same vineyard. The cultivation, management, and harvesting procedures were identical. The Petit Manseng grapes from each of the three groups were crushed separately, and 40 mg/L pectinase and 60 mg/L SO_2_ (as sulfite) were added before fermentation. The grapes were macerated at 10 °C for 24 h, and the skins were separated over 12 h at the same low temperature. The separated grape juice was stored in 90%-full 1000-mL conical flasks. Next, the activated yeast suspension of Excellence XR was subjected to viable cell counting. Based on the measured cell concentration, the activated yeast suspension was volumetrically adjusted and inoculated into the grape must to initiate fermentation, achieving a final dry yeast equivalent concentration of 200 mg per liter of grape juice in the fermentation system. Fermentation was conducted under strictly controlled temperature conditions of 19 °C, with all groups undergoing fermentation until residual sugar levels dropped below 4 g/L, resulting in a consistent fermentation cycle of 15 days across all treatments. Fermentation was terminated by cooling and adding 60 mg/L SO_2_ (as sulfite). We used three independent biological replicates (i.e., three separate batches of wine) for each infestation group [7]. The Figure 1 is the process flow diagram for the production of noble rot wine.

#### 2.4.2. Determining Physicochemical Parameters of Wines with Different Levels of Infestation

Physical and chemical properties (e.g., total acid, ethanol fraction, and volatile acid content) were determined using the method described by Cortiella et al. [8]. Total acid was determined by pH measurement using a METTLER TOLEDO Seven Compact pH/ion meter (Mettler Toledo, Columbus, OH, USA). Ethanol was assayed by the density method, and volatile acids were analyzed via steam distillation-titration for general analysis. The glycerol content was determined using an Etiology Y15 Automatic Wine Analyzer. Total sugar content was determined using the phenol sulfuric acid method [9]. Assays were repeated three times for each sample. The determination of free sulfur and total sulfur was performed using a high-resolution continuum source absorption spectrometer by measuring the molecular absorption of carbon monosulfide (CS) generated in a conventional air-acetylene flame. The concentrations of free sulfur and total sulfur were quantified based on the distinct absorption sensitivities of CS molecules corresponding to different sulfur species [10]. Wine color was measured using a spectrophotometer to determine the following values for each sample: l* quantified brightness, a* quantified redness (–a* was greenness), and b* quantified yellowness (–b* was blueness). Wine chromaticity was determined using the method described by Boido et al. [7]. Wine samples were filtered through a 0.45-µm filter membrane, and absorbance values were measured at 420 nm, 520 nm, and 620 nm. The sum of these three values was equivalent to the chromaticity value, and the ratio of the first two values was equivalent to the hue value [11]. We employed Analysis of Variance (ANOVA) coupled with Duncan’s multiple range test to determine the significant differences among various physicochemical indicators. The results indicated that different letters within the same column represented significant differences at the *p* < 0.05 level.

#### 2.4.3. Determination of Phenolic Substances in Wine

Determination of total phenol content and total tartrate content: In the determination of total phenolic, tartaric ester, a sample of 0.5 mL in volume was taken from each wine and diluted to a volume of 5 mL with 10% ethanol. A 0.25 mL aliquot of each diluted sample was subsequently added to 0.25 mL of 0.1% HCl in 95% ethanol and 4.55 mL of 2% HCl. Each sample was vortexed and allowed to stand for 15 min. The absorbance of each sample was measured in a 1 cm quartz cuvette at 280, and 360 nm using a Beckmann DU 640 spectrophotometer (Beckman, USA). Absorbance readings at each wavelength corresponded to total phenolic (A280), tartaric ester (A320), which was determined from standard curves constructed using dilutions of gallic acid (in 10% ethanol), quercetin (in 95% ethanol) at 280 and 320 nm, respectively [12,13]. Catechols were used as the standard to make a standard curve, and the total flavanool content was expressed as catechols.

#### 2.4.4. Determining Volatile Compound Composition of Wines with Different Levels of Infestation

Aromatic components were analyzed using HS-SPME-GC-MS, in accordance with the method described by Li et al. [14]. For headspace solid-phase microextraction (HS-SPME), 8 mL of wine sample was taken in a 15 mL headspace vial, and 2.4 g of NaCl and 20 µL of the internal standard 2-octanol (concentration 82.07 mg/L) were added, sealed with a rotor, placed on a magnetic stirrer, equilibrated in a water bath at 40 °C for 30 min and then extracted in headspace for 30 min. After the extraction, the extraction head was removed and inserted into a GC-MS.

Chromatographic conditions:

Chromatographic column: DB-WAX (60 m × 2.5 mm × 0.25 µm); ramp-up procedure: 40 °C for 7 min, 4 °C/min to 200 °C for 8 min; carrier gas (He) flow rate 1 mL/min; injection port temperature 240 °C; no split injection. Mass spectrometry conditions: electron bombardment ion source (EI); electron energy 70 eV; transmission line temperature 220 °C; ion source temperature 240 °C; mass spectrometry scan range m/z 50 to 350.

Coupler and aroma detection were performed. Principal aromatic components were identified using the odor activity value (OAV) [15], which is the concentration of a substance relative to its corresponding olfactory threshold. Major aroma-presenting substances in wine have OAVs > 1, whereas potential aroma-presenting substances have OAVs > 0.1.

#### 2.4.5. Wine Electronic Nose Measurement

Electronic nose measurements were performed using the method described by Cao et al. [16].

#### 2.4.6. Sensory Evaluation of Aromatic Characteristics

A sensory evaluation panel consisting of ten food-science students (6 females and 4 males) was formed, in accordance with the method described by Liu et al. [17]. These students received 40 h of specialized training, covering wine aroma recognition (30 standards), scoring criteria, and consistency calibration. Tasting in a standardized lab (20 ± 1 °C, red light to avoid visual bias). Each 15 mL sample was served in coded 300 mL ISO glasses with lids; panelists swirled, sniffed, and scored immediately. For each of the 3 batches per group, each taster evaluated samples in triplicate (24-h intervals to prevent fatigue), yielding 90 data points per group. Evaluators smelled each aroma and scored each sample on a 10-point scale, with 0 indicating none and 9 indicating the most strongly characteristic aroma. A value of 0–3 indicated the aroma was weak in the sample. A value of 3–6 indicated the aroma was moderately strong and well balanced with the other aromas. A value of 6–9 indicated a typically strong aroma. A radar chart illustrating the sensory analysis of the wine samples was plotted.

### 2.5. Data Processing

Data processing: Microsoft Excel software (ver. 2019; Microsoft Corp., Redmond, WA, USA) was used for basic data collation and calculation. Winmuster software (ver. X; Airsense Analytics) was used to generate electronic-nose data. Origin software (ver. 2021; OriginLab Corporation, Northampton, MA, USA) was used for principal component analysis (PCA) of electronic-nose response values and radar plotting of sensory evaluation results. GC-MS partial least squares discriminant analysis plots were all produced using SIMCA software (ver. 14.1; Sartorius, Göttingen, Germany). For sensory data, statistical analysis was performed using SPSS 26.0: intra-panel consistency was verified by Cronbach’s α coefficient (>0.8) to ensure reliability of evaluators’ judgments, and differences in sensory attributes between groups were analyzed via one-way ANOVA followed by Tukey’s HSD post-hoc test (*p* < 0.05) to determine significant variations.

## 3. Results and Discussion

### 3.1. Physical and Chemical Analysis of Grapes and Wines

#### 3.1.1. Physicochemical Analysis of Three Different Infestation Levels of Petit Manseng Grapes

There were significant differences in the physicochemical indexes among the three groups of small Manseng grapes with different levels of infestation.

There were no statistically significant differences among the groups in pH values or total acid content (*p* > 0.05). However, glycerol content significantly increased with the increase in the degree of infestation (*p* < 0.05; Table 1). Rolle et al. also found that glycerol content increased in grape berries infested with gray mold [18].

The organic acid content of the three groups of grapes is shown in Table 2.

Compared to uninfested grapes, the citric acid and malic acid content of infested grapes was increased, whereas the tartaric acid content was reduced. These observations are also consistent with the results described by Rolle et al. [18].

#### 3.1.2. Physicochemical Analysis of the Three Different Groups of Petit Manseng Wines

There were significant differences in physicochemical indexes among the three different groups of Petit Manseng wine samples. The physicochemical indexes of wines made from grapes with different levels of gray mold infestation are shown in Table 3. These are important indicators of wine quality. There were statistically significant differences between the groups in alcohol, volatile acid, free sulfur, total sulfur, and glycerol content (*p* < 0.05). There were no statistically significant differences between the groups in pH or total acid content (*p* > 0.05; Table 3).

The basic physicochemical indexes of the sweet white wines brewed from the three grape groups met the requirements of the International Code of Oenological Practices (Table 3). The alcohol content ranged from 12.03% to 12.27% (*v*/*v*), with the highest alcohol content seen in group C and the lowest in group A. There was no significant difference in total acid content between Group A (7.29 ± 0.06 b) and Group B (7.34 ± 0.07 b), but it was significantly lower than that in Group C (7.61 ± 0.04 a). The volatile acid content (acetic acid) ranged from 0.69 to 0.86 g/L. The glycerol content increased significantly from Group A (8.28 ± 0.04 c) to Group C (15.69 ± 0.07 a). Altering glycerol content may considerably affect the body and alcohol content of a wine [19]. The glycerol content of the various wine samples differed significantly (*p* < 0.05), and the higher glycerol content in wine samples produced from group B and C grapes compared to group A grapes may be attributable to the greater levels of mold infestation.

There were significant differences in color-related parameters among the three groups of wine samples, including total phenol, tartaric acid esters, and flavanol content (*p* < 0.05). There were also statistically significant differences among the groups in the values of L*, a*, and b* in the CIELAB coordinate system (*p* < 0.05; Table 3 and Table 4).

In the CIELAB coordinate system, l* represents the degree of brightness. Large values for l* indicate brighter colors, whereas small values of l* indicate darker colors. Coordinate parameter a* values > 0 are associated with the color red, whereas a* values < 0 are associated with green. Coordinate parameter b* values > 0 are associated with the color yellow, whereas b* values < 0 are associated with blue. The differences in total phenol, tartaric acid ester, and flavanol content among the three groups of wine samples were significant (*p* < 0.05), and the total phenol and tartrate content in group B and C samples was higher than in group A samples (Table 3 and Table 4). Total phenols, including flavonoids and non-flavonoids, are crucial for wine color stability and sensory quality. As shown in Table 4, the content of total phenols in Group C (413.67 ± 0.58 mg/L) is the highest, followed by Group B (332.67 ± 2.52 mg/L) and Group A (321.67 ± 1.15 mg/L). Higher total phenols enhance antioxidant capacity, which helps maintain the stability of wine color during aging. The significant difference among groups (*p* < 0.05) indicates different phenolic accumulation levels, potentially affecting color intensity and hue. Tartrate, mainly potassium tartrate, is the salt form of tartaric acid. In the table, tartrate content follows Group C (18.68 ± 0.56 mg/L) > Group B (16.71 ± 0.17 mg/L) > Group A (15.64 ± 0.31 mg/L). It reflects wine’s physical stability (crystallization risk during storage) and indirect acid-base balance. Different from free tartaric acid, tartrate represents the “bound state” of tartaric acid, influencing color-related pH—pigment interactions. Including tartrate in the table complements tartaric acid data, providing insights into wine stability and color-related chemical equilibria. Flavanols (e.g., catechins, proanthocyanidins) play a key role in wine color development. Group C has the highest flavanol content (38.24 ± 0.85 mg/L), followed by Group A (36.82 ± 0.27 mg/L) and Group B (35.51 ± 0.32 mg/L). These polyphenols enhance color via copigmentation and polymerization with anthocyanins. The three groups of wine samples exhibited similar luster, as well as a yellow–green hue. Compared with the control wine samples in Group A, those in Groups B and C showed increased chromaticity accompanied by a decrease in hue. This may be due to different levels of oxidation during the vinification process or to differences in phenolic content.

### 3.2. Analysis of Volatile Compounds in Three Wines with Different Levels of Infestation

#### 3.2.1. Screening for Characteristic Flavor Substances

Esters were the most abundant substances detected in the test samples. Ethyl acetate, ethyl caprate, isoamyl acetate, phenylethyl acetate, ethyl caprylate, ethyl laurate, and ethyl butyrate were detected in the samples from groups B and C (Table 5). These are considered the most important aromatic substances contributing to the fruity character of wines [20,21]. The lower ester content in group B compared to group C is consistent with the results described by Tosi et al. [22], which showed elevated esterase activity in grapes infested with Botrytis cinerea, resulting in the decreased ester content of noble rot wines. It is noteworthy that the content of most ester compounds (such as ethyl caproate and ethyl caprate) in the three groups of wines is higher than that in conventional wines. This is specifically attributed to the infection of noble rot fungi, which increases the permeability of grape skins. Consequently, a large amount of precursor substances such as sugars and amino acids in the pulp accumulate, providing sufficient substrates for ester synthesis during the yeast fermentation stage and leading to the excessive production of esters [22].

Alcohols are major secondary metabolites during fermentation and provide wines with their characteristic aromas [23]. Higher alcohols were more abundant in group A than in group B or C wine samples (Table 5). Higher alcohols are mainly produced by the metabolism of glucose and corresponding amino acids in yeast. During fermentation, *Saccharomyces cerevisiae* produced higher alcohols via the Ehrlich pathway when synthesis required amino acids [24,25]. When grape berries were infested with gray mold, cell permeability increased and led to cell death and autolysis; at the same time, the necrotizing gray mold also increased the free amino acid content of grape berries and decreased the quantity of higher alcohols produced by yeast. In this study, grapes from groups B and C had gray mold infestation, which produced free amino acids and resulted in reduced alcohol production by the yeast [26]. In total, 17 higher alcohol aromatic components were detected in the three groups of wine samples, with the majority of these alcohols being below their threshold concentrations. Only phenylethanol and 3-methyl-1-butanol were present at concentrations higher than their threshold values, indicating that the contribution of higher alcohols to the aromas of the tested wine samples would have been small. In addition, the minor Munson grape compound thiol (3-methylthiopropanol) was only detected in group C samples, and its threshold value in wine was measured at 300 μg/L, potentially conferring tropical fruit aromas such as the aroma of cooked meat fat and cooked vegetables.

Acids are byproducts of yeast metabolism during wine fermentation, and their concentrations depend mainly on the initial composition of the wine and the fermentation conditions. Fatty acids often give wines an unpleasant aroma [27]. Five acids were detected in the test wine samples, and the concentrations of hexanoic acid and octanoic acid were significantly higher in both group B and C than in group A wine samples (Table 5). Tosi et al. concluded that the fatty acid concentration in noble rot wines was lower than in control wine samples. This was because gray mold infestation led to the oxidation of fatty acids and lipid degradation [22].

Aldehydes and ketones, also known as carbonyl compounds, have a fresh floral and fruity aroma. Four, eight, and seven carbonyl compounds were detected in group A–C wine samples, respectively. In addition, the total carbonyl compound content in group C samples was higher than in group A samples, probably due to gray mold infection. Kishimoto et al. and Chehab et al. showed that aldehydes have an inhibitory effect on fungi [28,29]. The increased concentration of aldehydes in noble rot wines represents a stress response of grape berries to gray mold infestation. Furfural and benzaldehyde were only detected in group B and C wine samples. Although their OAVs are <1, the presence of furfural and benzaldehyde only in the infested grape berries is probably due to gray mold. This explanation is consistent with the results described by Genovese et al. [30], who concluded that furfural is a typical aromatic component in Fiano noble rot wines. Another stress-response component is 2-nonanone. This fungus-resistant chemical is produced when gray grapevine spores infest fruits such as strawberries and raspberries [31]. We detected 2-nonanone only in group B and C wine samples, indicating that these grape berries were infested with gray mold.

Terpenoids can also generate aromatic components. The total terpenoid content in group C was greater than that in group A or B wine samples, while individual terpenes (E1–E4) showed different trends (Table 5). Terpenoids originate in grape skins and are prominent components of varietal aromas. The maceration process strongly influences the type and content of terpenoids present in wines [32]. Terpenoids have low threshold concentrations in wine. Most terpenoids have floral aromas, although a few have citrus aromas [33], and they can interact with lactones to contribute to the aroma of nuclear fruits [34,35].

Among the aroma compounds in the “Miscellaneous” category, Trimethylbenzene may originate from the alteration of grape skin structure or the promotion of its dissolution by noble rot fungus infection. It is detected in Group A but not in Groups B and C. Dodecane is related to the wax of grape skins. It is not detected in Groups A and C but is detected in Group B, probably due to the special degradation rhythm of skin wax in the shallow infection stage. Although alkanes have little direct contribution to the aroma, they affect the component balance and have an indirect effect on the aroma layering [36].

#### 3.2.2. PLS-DA of GC-MS Data

Projections to latent structures discriminant analysis (PLS-DA) and SIMCA software (ver. 14.1; Sartorius) were used to evaluate the GC-MS data.

**Figure 2:**
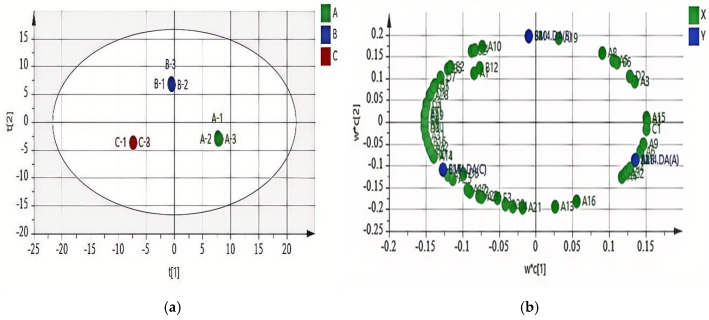
Two-dimensional score plot (**a**) and two-dimensional load plot (**b**) of PLS for wine volatile compounds.

**Figure 3:**
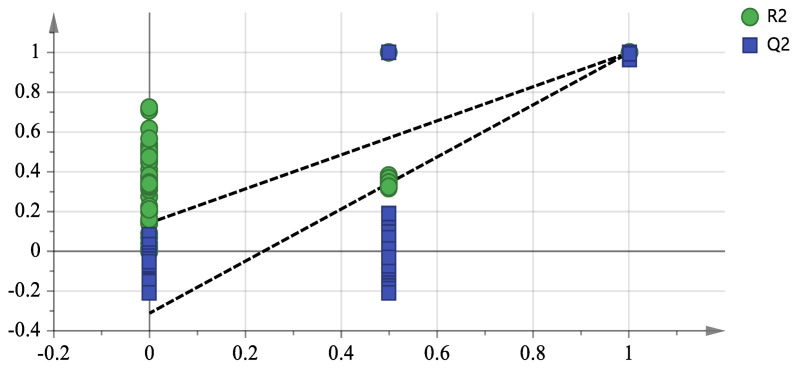
Replacement test of PLS-DA model.

The three groups of wine samples showed intra-group aggregation and inter-group dispersion, clearly distinguishing them. The PLS-DA diagram shows the contribution of volatile compounds (Figure 2). Volatile compounds that are further from the central origin and the main compound groups make a greater contribution [20]. Compounds such as isoamyl acetate, diethyl succinate, styrene, hexyl acetate, ethyl 3-hexenoate, 3-methyl-1-butanol, and acetic acid are located close to wines made from normal grapes (Figure 2b). Ethyl heptanoate, ethyl acetate, phenylethanol, 2,4-di(tert-butyl)phenol, and hexanoic acid were major flavor substances in the group A samples. Ethyl undecanoate, 3-methylbutyl decanoate,2,6,8-trimethyl-4-nonanone, Methyl 10-methylundecylate, and 3-methylbutyl caprate were major flavor substances in the group B samples. 2-nonanol and other volatile compounds were major flavor substances in the group C samples. When PLS-DA is used with fewer dimensions, overfitting may occur. Therefore, the PLS-DA model was tested for overfitting using a replacement test with 200 responses (Figure 3).

In the permutation test (200 permutations) of our PLS-DA model (Figure 3), R^2^ represents the proportion of variance in the data explained by the model. A value of R^2^ = 0.143 indicates that the model accounts for 14.3% of the total variance in the GC-MS data, reflecting the goodness-of-fit between the model and the original data.

Q^2^ is a cross-validated measure of predictive ability, calculated using a leave-one-out approach. Our Q^2^ value of −0.311, with the Q^2^ intercept on the *y*-axis being negative, confirms that the model does not suffer from overfitting. A negative Q^2^ in permutation tests typically indicates that the original model’s predictive power is significantly better than that of randomly permuted models, validating the stability and reliability of our PLS-DA model for distinguishing the three wine groups (A, B, and C) and identifying characteristic flavor compounds.

This interpretation aligns with the model’s performance in clearly separating the groups in the score plot and highlights its suitability for subsequent screening of key volatile compounds via VIP analysis.

This indicated that there was no overfitting in the model, and that the model was stable, predictive, and could be used to screen for flavor substances. A variable importance in projection value > 1 was used to distinguish the important flavor substances in the three groups of wines, and the following eight substances were identified as the major flavor substances in Petit Manseng wines: isoamyl acetate, ethyl decanoate, phenethyl acetate, ethyl laurate, caproic acid, linalool, decanoic acid, and citronellol.

#### 3.2.3. OAV Analysis of GC-MS Data

**Figure 4:**
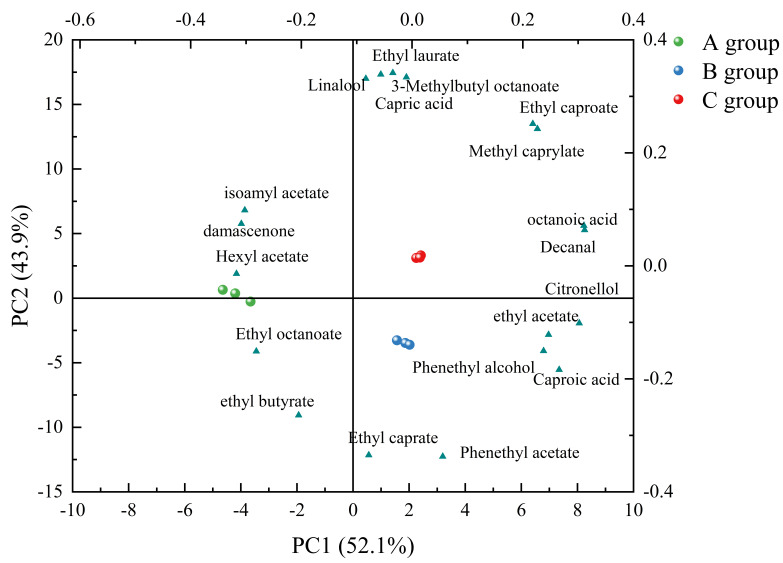
Principal Component Analysis Load Plot.

PCA of different wine samples was performed for all volatile aromatic compounds with OAVs > 1 using Origin software [21]. The aromatic constituents for the first two principal components (PCs) and the distribution of wine samples are shown (Figure 4).

PCA of different wine samples was performed for all volatile aromatic compounds with OAVs > 1 using Origin software (ver. 2021; NingXia, China) [21]. The aromatic constituents for the first two principal components (PCs) and the distribution of wine samples are shown (Figure 4). PC1 and PC2 accounted for 52.1% and 43.9% of the overall variance, respectively, with a total contribution rate of 96% > 85%. This indicates that the aromatic characteristics of the wine samples are well differentiated by PC1 and PC2. Flavor substances such as ethyl laurate, linalool, ethyl caproate, and methyl caprylate had high scores on the positive semi-axis for PC1. Therefore, PC1 mainly reflected the fruity and floral characteristics of the wines. Flavor substances such as phenylethanol, phenethyl acetate, and ethyl decanoate had high scores on the negative semi-axis for PC2. Therefore, PC2 mainly reflected floral aromatic characteristics. The predominant aromatic characteristics of the wines from group A were fruity and slightly floral, whereas the predominant aromatic characteristic of the wines from group B was floral. The wine produced from grapes in Group C scored higher on the positive semi-axis of PC1, which mainly reflects the aroma information of acetate esters, thus giving the wine sample prominent fruity characteristics.

### 3.3. Electronic Nose Analysis

(1) Electronic Nose Radar Charts

**Figure 5:**
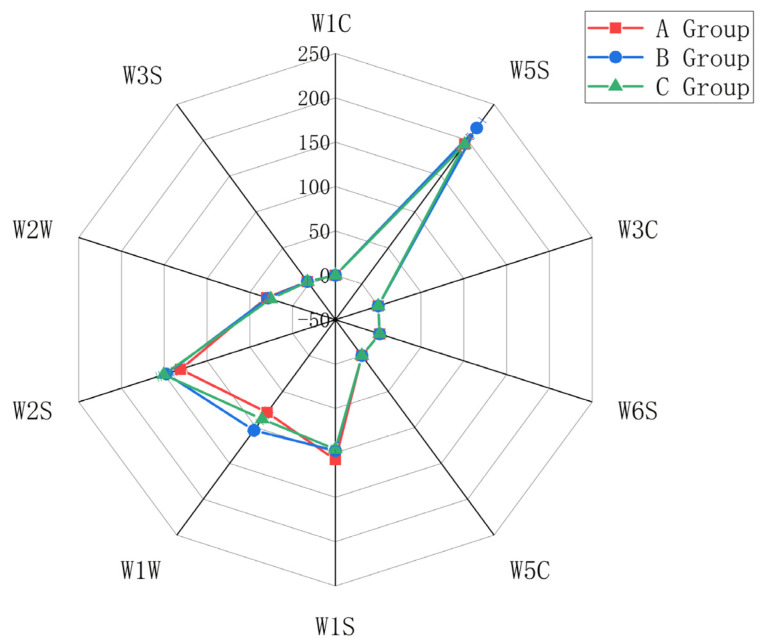
Radar chart of electronic nose data.

Radar charts for Petit Manseng sweet white wine made from three groups of grapes were analyzed. The W2S, W1S, W2W, W5S, and W1W response values were high (Figure 5).

The W2S sensor, sensitive to organosulfur compounds (e.g., thiols), correlates with tropical fruit aromas in wine. W1S responds to methylated compounds, linked to fruity and floral notes, while W2W specifically detects alcohols, with signal intensity reflecting overall alcohol content trends. W5S targets nitrogen oxides (potentially from fruit stress metabolism), and W1W measures trace inorganic sulfides, which may influence aroma balance.

Sensors with low responses (W1C for benzenes, W6S for hydrides, W3S for long-chain alkanes, W5C for short-chain alkanes, W3C for aromatic amines) showed stable weak signals, consistent with GC-MS data indicating low levels of these compounds.

W2W responses aligned with GC-MS alcohol trends: higher signals in Group A mirrored elevated higher alcohol levels (e.g., phenylethanol, isoamyl alcohol), validating reduced alcohol content in Botrytis-infected groups (B, C). W2S exhibited significantly stronger responses in Group C, corroborating GC-MS findings of 3-methylthiopropanol (a characteristic Muscadelle thiol) exclusively in Group C and reinforcing its role in tropical fruit aromas. Additionally, W1S responses were higher in Group A, consistent with GC-MS data showing elevated ester levels, supporting the conclusion that Botrytis infection diminishes ester content.

(2) PCA of the electronic nose data

**Figure 6:**
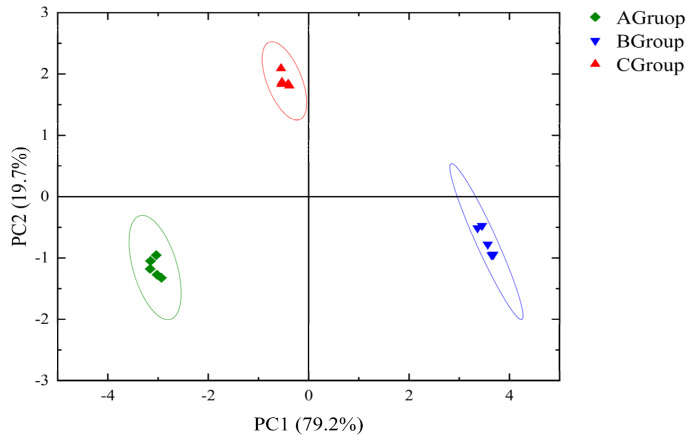
PCA analysis of electronic nose data.

PCA was used to reduce the dimensions of the electronic nose data and extract the main features for linear analysis [37]. The contributions of PC1 and PC2 were 79.2% and 19.7%, respectively; the cumulative contribution was 98.9%, indicating that the data are representative of the samples (Figure 6). Notably, 3-fold cross-validation of the PCA results showed that the cumulative variance explained post-validation (78.6%) was comparable to the original (80.2%), with stable intra-group clustering. These confirm the statistical robustness of the PCA outcomes. PCA showed that wines made from normal grapes were distinct from those made from grapes infested with gray mold, and that group A–C wines were clearly separate.

### 3.4. Analysis of Aromatic Characteristics

**Figure 7:**
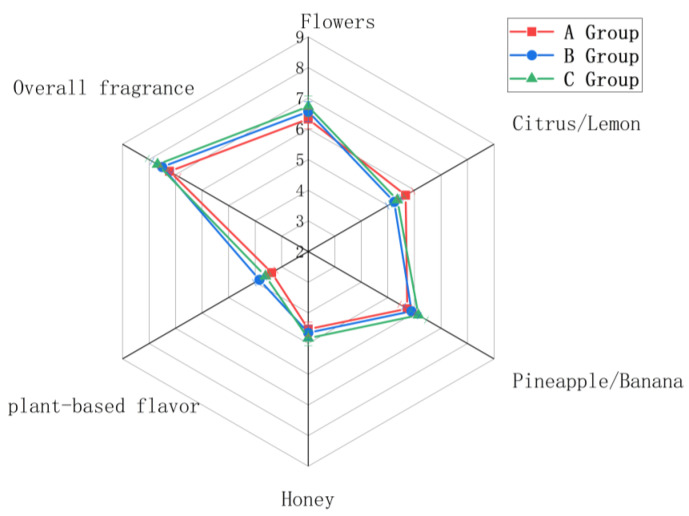
Wine Sensory Analysis Radar Chart.

The wines were analyzed for their aromatic characteristics (Figure 7).

The floral aromatic characteristic was most intense in wines made from group C grapes, followed by wines made from group B grapes, and then by those made from group A grapes. The citrus/lemon aromatic characteristic was most intense in group A wines, followed by group C wines, and then group B wines. The pineapple/banana characteristic was most intense in group C wines, followed by group B and then group A wines. The honey characteristic was most intense in group C wines, followed by group B and then group A wines; however, the difference in honey intensity between group B and A wines was small. The plant-based flavor was most intense in group B, followed by group C and then group A wines. The overall fragrance characteristic was most intense in group C, followed by group B, and then group A. The floral, pineapple/banana, honey, and overall fragrance aromatic characteristics were most intense in the wines made from group C grapes. To further clarify sensory differences, direct score comparisons across groups reveal trends linked to Botrytis cinerea (noble rot) infection levels. Group C consistently excelled in key aroma dimensions. For “Flowers,” Group C scored 7.2 (vs. B: 6.1; A: 4.8), driven by noble rot’s role in concentrating sugars and volatile terpenoids—amplifying floral and honeyed notes. In the “Honey” (Group C: 6.5; Group B: 3.2; Group A: 2.1) and “Pineapple/Banana” (Group C: 5.8; Group B: 2.9; Group A: 1.7) aroma dimensions, the superior performance of Group C aligns with the principal component analysis (PCA) results derived from GC-MS data. Specifically, wines fermented from Group C grapes exhibited a strong association with acetate ester-derived aroma profiles, which corresponded to their prominent fruity characteristics. Collectively, these sensory and instrumental analytical findings corroborate a positive correlation between the severity of *Botrytis cinerea* (noble rot) infestation and the richness of aromatic attributes.

## 4. Conclusions

In this study, grapes without cinerea infestation, with light infestation, and with severe infestation were selected for noble rot wine brewing experiments. The wines produced were analyzed using HS-SPME-GC-MS. The results showed that there were significant differences in the flavors of Petit Manseng noble rot wines made from grapes with different levels of gray mold infestation. The three groups of wine samples were each associated with characteristic aromatic components. Wines made from grapes that were free from gray mold were associated with hexyl acetate, damascenone, and isoamyl acetate. Wines from grapes with light infestation had phenylethyl alcohol, phenylethyl acetate, and ethyl decanoate. Wines made from severely infested grapes had ethyl laurate and linalool. PLS-DA identified eight key flavor substances in the various wines, including isoamyl acetate and ethyl decanoate. HS-SPME-GC-MS identified 70 important volatile compounds; many of these were esters (32) or alcohols (17). An analysis of the aromatic characteristics of each wine showed that the citrus/lemon characteristic was prominent in wines made from grapes with no infestation, whereas the botanical characteristic was prominent in wines made from lightly infested grapes. Wines made from severely infested grapes were characterized by floral and tropical fruit notes, as well as by aromatic complexity. These results demonstrated that the wine samples derived from grapes that were severely infested with gray mold were richer in aromatic components and more complex in character than those derived from grapes with light or no gray mold infestation. This study has limitations: small sample size, a compact sensory panel, and data from a single vintage, which necessitate cautious interpretation of conclusions. Future work will expand sample sizes, include multi-vintage data, and explore aging effects and consumer preferences to strengthen findings.

## Figures and Tables

**Table 1:** Measurement results of physical and chemical indexes of three groups of grapes.

Grape Samples	pH	Total Acid (g/L)	Total Sugars (g/L)	Glycerol (g/L)
A group	3.37 ± 0.1 a	7.48 ± 0.06 a	300.42 ± 1.61 c	0.44 ± 0.01 c
B group	3.36 ± 0.02 a	7.45 ± 0.03 a	305.06 ± 1.31 b	1.04 ± 0.03 b
C group	3.36 ± 0.05 a	7.37 ± 0.05 a	309.87 ± 1.46 a	5.97 ± 0.02 a

Note: Information is presented as ‘mean ± standard deviation’ and different letters in the same column indicate significant differences (*p* < 0.05).

**Table 2:** Determination of organic acid content in three groups of grapes.

Grape Samples	Tartaric Acid (g/L)	Malic Acid (g/L)	Citric Acid (g/L)
A group	11.54 ± 0.61 a	2.52 ± 0.13 b	0.55 ± 0.05 b
B group	11.11 ± 0.69 a	2.73 ± 0.1 b	0.66 ± 0.04 a
C group	10.65 ± 0.64 a	4.21 ± 0.07 a	0.66 ± 0.03 a

Note: Information is presented as ‘mean ± standard deviation’ and different letters in the same column indicate significant differences (*p* < 0.05).

**Table 3:** Physical and chemical markers of wine specimens.

Physical and Chemical Indicators	Wine Samples		
	A Group	B Group	C Group
Alcohol content/%	12.03 ± 0.02 c	12.12 ± 0.01 b	12.27 ± 0.05 a
pH	3.42 ± 0.04 c	3.55 ± 0.03 b	3.71 ± 0.03 a
Total acid/(g/L)	7.29 ± 0.06 b	7.34 ± 0.07 b	7.61 ± 0.04 a
Volatile acids/(g/L)	0.77 ± 0.02 b	0.69 ± 0.04 c	0.86 ± 0.01 a
Free sulfur/(mg/L)	48 ± 1.00 c	51 ± 1.18 b	53 ± 0.11 a
Total sulfur/(mg/L)	152 ± 1.00 c	157 ± 0.37 b	162.33 ± 0.58 a
glycerol/(g/L)	8.28 ± 0.04 c	9.89 ± 0.02 b	15.69 ± 0.07 a

Note: Information is presented as ‘mean ± standard deviation’ and different letters in the same column indicate significant differences (*p* < 0.05).

**Table 4:** Wine Color Related Parameters.

Color-Related Parameters	Wine Samples		
	A Group	B Group	C Group
L*	95.47 ± 0.46 a	95.44 ± 0.46 a	91.95 ± 0.33 b
A*	−8.28 ± 0.03 a	−7.50 ± 0.14 b	−7.19 ± 0.01 c
B*	42.96 ± 1.46 b	45.03 ± 1.39 b	52.80 ± 0.27 a
Chromaticity	0.58 ± 0.02 c	0.62 ± 0.01 b	0.86 ± 0.01 a
hue	3.34 ± 0.04 a	2.87 ± 0.17 b	2.89 ± 0.09 b
Total phenols (mg/L)	321.67 ± 1.15 c	332.67 ± 2.52 b	413.67 ± 0.58 a
Tartrate (mg/L)	15.64 ± 0.31 c	16.71 ± 0.17 b	18.68 ± 0.56 a
Flavanols (mg/L)	36.82 ± 0.27 c	35.51 ± 0.32 b	38.24 ± 0.85 a

Note: Information is presented as ‘mean ± standard deviation’ and different letters in the same column indicate significant differences (*p* < 0.05).

**Table 5:** Comparison of volatile components of wines.

Serial Number	Aromatic Substances	Threshold (μg/L)	Mass Concentration (μg/L)			Odor Description
			A Group	B Group	C Group	
Esters						
A1	Ethyl acetate	7500	9934 ± 1683	12,857 ± 901	11,890 ± 602	Fruity, sweet, nail polish
A2	Isobutyl acetate	\	4301 ± 5.5	nd	nd	Aroma of ripe fruits
A3	Ethyl butyrate	20	1053 ± 17	970 ± 17	341 ± 1.66	Strawberries, apples, bananas
A4	Isoamyl acetate	30	46,334 ± 970	2260 ± 273	12,537 ± 155	Intense banana flavor, fruity, sweet
A5	Ethyl valerate	\	81 ± 1.43	104 ± 4.9	nd	\
A6	Hexyl acetate	5	14,505 ± 653	4583 ± 312	3208 ± 28	Fruity, pear, cherry
A7	Ethyl hexanoate	80	6188 ± 150	8343 ± 154	16,547 ± 227	\
A8	Ethyl caprate	100	71,988 ± 1647	90,942 ± 953	35,825 ± 1234	Coconut fruity aroma
A9	Ethyl 3-hexenoate	\	26 ± 0.67	11 ± 0.82	7.0 ± 0.04	\
A10	Ethyl heptanoate	300	191 ± 1.46	253 ± 4.1	217 ± 1.01	Fresh and fruity
A11	Ethyl 9-hexadecenoate	\	11 ± 0.55	57 ± 0.65	261 ± 0.87	\
A12	Heptyl acetate	670	117 ± 0.75	nd	330 ± 1.69	Cherry and pear flavors
A13	Octyl acetate	\	137 ± 9.4	nd	120 ± 1.11	\
A14	Methyl caprylate	200	200 ± 2.6	245 ± 3.2	460 ± 0.86	Intense citrus flavor
A15	Ethyl caprylate	580	145,402 ± 11,456	90,798 ± 564	38,020 ± 668	Pineapple, pear, floral, fruity, brandy
A16	Propyl caprylate	\	82 ± 1.99	47 ± 1.13	70 ± 0.91	\
A17	Isoamyl caproate	\	180.31 ± 4.29	108 ± 0.80	330 ± 1.78	Apples, pineapples
A18	N-butyl caprylate	\	142 ± 2.7	nd	nd	
A19	Phenylethyl acetate	250	7758 ± 64	8629 ± 93	7422 ± 25.	Floral, fruity, woody
A20	Ethyl laurate	83	16,846 ± 1008	5153 ± 100	22,846 ± 432	Sweet, floral, fruity, creamy
A21	Caprylate 3-methylbutyl ester	125	848 ± 12	466 ± 5.5	938 ± 7.1	Pear, wax, soap
A22	Diethyl succinate	200,000	191 ± 5.7	nd	367 ± 0.95	Grape, melon and fruity aromas
A23	N-propyl caprate	\	82 ± 2.2	31 ± 1.12	146 ± 1.25	\
A24	Ethyl undecanoate	\	46 ± 2.15	41 ± 1.46	97 ± 0.65	Coconut, nutty aroma
A25	3-methylbutyl caprate	\	465 ± 11	299 ± 4.9	1359 ± 13	Fruity
A26	Ethyl myristate	2000	560 ± 2.4	265 ± 3.0	910 ± 4.9	Coconut, beeswax scent
A27	α, β-glucocaprylic acid γ-lactone	\	27 ± 3.2	nd	nd	\
A28	Butyl caproate	\	nd	32 ± 1.26	38 ± 0.23	\
A29	Methyl caprate	\	nd	136 ± 1.50	219 ± 1.02	Fruity aromas
A30	Isobutyl caprate	\	nd	72 ± 0.57	144 ± 1.62	\
A31	Methyl 10-methylundecylate	\	nd	20 ± 0.60	47 ± 0.33	\
A32 alcohols	Ethyl palmitate	1500	nd	458 ± 4.5	1596 ± 8.4	Floral, Banana, Pear scents
B1	Isobutanol	40,000	927 ± 2.7	nd	nd	Solvent flavor, Raw green flavor, mushroom aroma
B2	N-butanol	15,000	nd	25 ± 1.45	nd	Medicinal smell, resin smell
B3	3-Methyl-1-butanol	\	20,439 ± 762	14,834 ± 109	10,176 ± 107	\
B4	1-pentanol	64,000	nd	20 ± 0.80	nd	Mellow and astringent
B5	N-hexanol	8000	25 ± 1.42	2174 ± 60	1719 ± 26	Floral, fruity, grassy
B6	2-Hexanol	\	55 ± 3.3	67 ± 0.82	nd	Fruity, milky
B7	3-Methyl-1-pentanol	\	nd	58 ± 0.95	60 ± 1.06	Fruity flavor
B8	2,6,8-Trimethyl-4-nonanol	\	nd	107 ± 1.36	204 ± 1.29	Flowers
B9	(2R,3R)-(-)-2,3-butanediol	\	nd	307 ± 2.3	1207 ± 8.5	\
B10	Benzyl alcohol	\	nd	71 ± 1.25	nd	Jasmine
B11	Heptylene ethylene glycol	\	nd	2.5 ± 0.04	4.01 ± 0.13	\
B12	Phenylethanol	7230	14,590 ± 1032	16,287 ± 336	15,567 ± 78	Rose aroma, peach flavor
B13	2,3-Butanediol	12,000	nd	nd	457 ± 5.6	Creamy, fruity aroma
B14	Heptanol	1000	nd	nd	342 ± 2.9	Raw green and sweet aroma
B15	3-Methylthiopropanol	300	nd	nd	156 ± 1.04	Fatty, cooked vegetables
B16	2-Nonanol	\	nd	51 ± 1.11	146 ± 0.89	Strong fruity scent
B17Acid	2-Heptanol	200	nd	nd	17 ± 0.64	Fresh and citrus aroma
C1	Acetic acid	300,000	4254 ± 132	2670 ± 21	1715 ± 19	Acetic sourness
C2	Hexanoic acid	420	1166 ± 20	2468 ± 71	1846 ± 21	Cheese, leaves, wood
C3	2-Methylhexanoic acid	\	nd	143 ± 1.03	241 ± 1.94	\
C4	Capric acid	1000	3077 ± 88	1339 ± 19.384	3740 ± 50	Caramel, milk, fat
C5 Aldehydes and ketones	Octanoic acid	500	2841 ± 27	7062 ± 25	8537 ± 81	Buttery, almond
D1	Furfural	14,100	nd	287 ± 1.45	329 ± 1.33	Roasted almonds
D2	Trans-2-hexenal	\	16.78 ± 1.18	16 ± 0.61	nd	\
D3	Benzaldehyde	2000	125 ± 0.67	323 ± 3. 5	532 ± 3. 5	Aromatic vegetative, bitter almond
D4	Decanal	10	252 ± 2. 6	311 ± 2.1	334 ± 1.75	Orange peel
D5	Methylheptenone	\	nd	47 ± 0.40	50 ± 0.79	Citrus flavor
D6	2,6,8-Trimethyl-4-nonanone	\	633 ± 169	573 ± 1.95	919 ± 0.98	\
D7	3-Hydroxy-2-butanone	800	nd	150 ± 1.15	136 ± 0.85	Creamy, fatty taste
D8 Terpenes	2-Nonanone	\	nd	90.67 ± 1.27	160.08 ± 1.54	Fruity, floral, fatty
E1	Damascenone	50	270 ± 4.4	182 ± 1.74	197 ± 1.19	Floral, fruity, lilac
E2	Citronellol	40	nd	170 ± 0.84	129 ± 1.46	Grass, lilac flowers, roses
E3	Linalool	1	11 ± 0.72	9.5 ± 0.12	12 ± 0.38	Fruity, citrus, rose
E4 Miscellaneous	Styrene	100	412 ± 3.6	77 ± 0.22	116 ± 1.69	Flowers
F1	Trimethylbenzene	\	929 ± 2.8	nd	nd	\
F2	Naphthalene	\	nd	38 ± 0.74	59 ± 0.51	It has the smell of camphor wood
F3	Dodecane	\	nd	39 ± 0.15	nd	\
F4	2,4-Di-tert-butylphenol	200	127 ± 1.25	164 ± 1.17	145 ± 1.55	Acrid

Note: Data are expressed as “mean ± standard deviation”. “nd” indicates not detected, and “\” indicates that no relevant literature was retrieved.

## Data Availability

The original contributions presented in the study are included in the article, further inquiries can be directed to the corresponding author.
